# Exact Inference for Random Effects Meta-Analyses for Small, Sparse Data

**DOI:** 10.3390/stats8010005

**Published:** 2025-01-07

**Authors:** Jessica Gronsbell, Zachary R. McCaw, Timothy Regis, Lu Tian

**Affiliations:** 1 Department of Statistical Sciences, University of Toronto, Torronto, ON M5S 1A1, Canada;; 2 Department of Biostatistics, University of North Carolina at Chapel Hill, Chapel Hill, NC 27599, USA;; 3 Department of Biomedical Data Science, Stanford University, Stanford, CA 94305, USA;

**Keywords:** exact inference, meta-analysis, random effects model, rare events, rosiglitazone

## Abstract

Meta-analysis aggregates information across related studies to provide more reliable statistical inference and has been a vital tool for assessing the safety and efficacy of many high-profile pharmaceutical products. A key challenge in conducting a meta-analysis is that the number of related studies is typically small. Applying classical methods that are asymptotic in the number of studies can compromise the validity of inference, particularly when heterogeneity across studies is present. Moreover, serious adverse events are often rare and can result in one or more studies with no events in at least one study arm. Practitioners remove studies in which no events have occurred in one or both arms or apply arbitrary continuity corrections (e.g., adding one event to arms with zero events) to stabilize or define effect estimates in such settings, which can further invalidate subsequent inference. To address these significant practical issues, we introduce an exact inference method for random effects meta-analysis of a treatment effect in the two-sample setting with rare events, which we coin “XRRmeta”. In contrast to existing methods, XRRmeta provides valid inference for meta-analysis in the presence of between-study heterogeneity and when the event rates, number of studies, and/or the within-study sample sizes are small. Extensive numerical studies indicate that XRRmeta does not yield overly conservative inference. We apply our proposed method to two real-data examples using our open-source R package.

## 1. Introduction

Meta-analysis is widely used in clinical research to aggregate information from similar studies to yield more efficient inference and improve statistical power [1–3]. It is particularly useful for assessing the frequency of adverse events in drug safety studies as single studies are typically powered to establish treatment efficacy and adverse events are rare. However, the validity of most existing meta-analytic approaches rests on the asymptotic distribution of the combined point estimator, which can be unreliable when any of the following conditions hold: (i) the event rates are low, (ii) the number of studies is not large, and (iii) the study-specific sample sizes are small [4–7]. This is a significant practical issue, as these conditions are common within the literature. A study of 500 Cochrane systematic reviews found that 50% of drug safety meta-analyses contained an outcome with a rare event rate (<5%) and 30% contained at least one study with no events in one arm [8]. Additionally, only 16% of meta-analyses considered had four or more studies. A well-known and controversial example where these issues arise involves the Type II diabetes drug, rosiglitazone, which was suspected to increase the risk of myocardial infarction (MI) and cardiovascular death (CVD) [9]. The available data are presented in Supplementary Table S1 and provided an initial motivation for this work.

In the original meta-analysis of the rosiglitazone data, the authors utilized the conventional fixed effect Peto method based on the combined odds ratio [9]. The authors found that rosiglitazone was associated with a significant increase in the risk of myocardial infarction (odds ratio, 1.43; 95% confidence interval, 1.03 to 1.98) and a borderline-significant finding for death from cardiovascular causes (odds ratio, 1.64; 95% confidence interval, 0.98 to 2.74). This finding set off significant debate over the drug’s safety, influencing regulatory actions worldwide, including label warnings by agencies like the Food and Drug Administration [10]. Because the events of interest were extremely rare, 25 of the 48 studies had no CVD and 10 studies had no MIs. The authors excluded these double-zero (DZ) studies (i.e., studies with no events on both treatment arms) from their analysis. Much discussion has since ensued regarding conflicting conclusions of alternative analyses of the rosiglitazone data based on the widely used fixed-effect Mantel–Haenszel method, which similarly relies on a normality approximation and requires removal of DZ studies or use of continuity corrections (e.g., adding one event to arms with zero events) [10–12].

As there is no clear guidance for removing studies or applying continuity corrections, Tian et al. proposed an exact confidence interval (CI) for fixed-effect meta-analyses based on the combination of study-specific exact CIs, which utilizes all available data and avoids continuity corrections [6,13,14]. Liu et al. later extended this approach with a method that combines p-value functions using the mid-p adaptation of Fisher’s exact method [15]. While these procedures are more robust than their classical counterparts, the underlying fixed effect assumption implies that all study-specific treatment effects are identical. In the case of the rosiglitazone study, for example, this assumption is likely violated as the studies had different eligibility criteria, medication doses, control and concomitant medications, and follow-up times [16].

An alternative and less restrictive approach is to employ a random effects analysis [17]. The most popular procedure is the DerSimonian–Laird (DL) method [18]. The DL combined point estimator is a linear combination of study-specific estimates of the effect of interest with weights based on the within- and between-study variation estimates. Depending on the chosen effect measure, similar issues arise with respect to continuity corrections or removal of DZ studies, making the application of the DL method to the rosiglitazone data questionable [19–21]. Moreover, the DL method relies on the assumption that the study-specific effects follow a normal distribution, which is unlikely to hold in the rare events setting. The validity of inference based on the DL method is further threatened when the number of studies is small, as the between-study variance is imprecisely estimated [22]. Difficulties in estimating the between-study variance have similarly limited the application of random effects regression-based approaches, particularly in the rare event setting [23]. To overcome these challenges, Shuster et al. introduced a ratio estimator for random effects meta-analysis for the setting of low event rates leveraging results from sampling theory [16]. Their findings differed from the original meta-analysis of the rosiglitazone data, indicating an elevated risk of CVD and no increased risk of MI with the use of the medication. Cai et al. later developed a likelihood-based approach based on a Poisson random effects model [7]. In contrast to the previously proposed ratio estimator, the authors employed a conditional inference argument to avoid continuity corrections and theoretically justified exclusion of DZ studies. Jiang et al. also introduced a method for obtaining profile confidence limits for the risk difference using importance sampling [24]. While these methods all target the setting with low event rates, the proposed inference procedures remain asymptotic in the number of studies and, to the best of our knowledge, are not available in open-source software. More recently, Zabriskie et al. proposed a permutation-based approach based on conditional logistic regression [25]. However, this method cannot be applied to all datasets with low event rates, does not uniformly guarantee Type I error control, and is computationally intensive.

The majority of existing random-effects meta-analyses therefore primarily accommodate datasets with a large number of studies, sufficiently high event rates, and/or large within-study sample sizes. To fill the current methodological gap in accommodating meta-analyses with small and/or sparse datasets, we introduce an exact inference procedure for random effects meta-analysis of a treatment effect in the two-sample setting with rare events, which we coin “XRRmeta”. XRRmeta is designed for meta-analyses of rare event outcome data, as it is based on a conditional inference argument that justifies the removal of DZ studies. Moreover, XRRmeta yields a CI through inversion of exact tests and is therefore guaranteed to achieve coverage at or above the nominal level (up to Monte Carlo error) regardless of the number of studies and/or within-study sample sizes. Importantly, our numerical studies indicate that our choice of test statistic yields inference that is not overly conservative and enables us to develop a procedure that is computationally feasible to run on a personal laptop computer. XRRmeta is also available in open-source R software at https://github.com/zrmacc/RareEventsMeta (accessed on 16 October 2024) to encourage its use in practice.

The remainder of this paper is organized as follows. In Sections 2 and 3, we present the methodological and computational details of XRRmeta. The performance of XRRmeta is then evaluated with extensive simulation studies in Section 4.1. In Section 4.2, we apply our procedure to the rosiglitazone study and a recent meta-analysis of face mask use in preventing person-to-person transmission of severe acute respiratory syndrome coronavirus 2 (SARS-CoV-2) and coronavirus disease 2019 (COVID-19). We close with additional remarks and avenues for future research in Section 5.

## 2. Problem Setup

### 2.1. Notations and Assumptions

Our goal is to compare the rates of an event of interest from multiple studies comparing the same treatments. We let $Y_{ij}$ be the number of events out of $N_{ij}$ subjects in the ith study and jth treatment arm and $K_{\text{tot}}$ be the total number of studies. The observed data consist of

$${𝒟}^{0}=\left\{\left(Y_{ij},N_{ij}\right)|i=1,\ldots K_{tot}\;\text{and}\;j=1,2\right\}.$$

Throughout, we let arm 1 correspond to the treated group and arm 2 correspond to the control group. As the $Y_{ij}$ are counts, we assume that they follow a Poisson distribution with the rate parameter following a log-linear model [7]. That is,

$$Y_{ij}\sim \text{Poisson}\left(N_{ij}\lambda_{ij}\right)$$

where $\lambda_{ij}=\lambda_{i2}e^{X_{ij}\xi_{i}},X_{ij}=I(j=1)$ is a binary indicator for assignment to the treated arm, $\lambda_{ij}$ is the event rate in the jth treatment arm of the ith study, and $\xi_{i}=\text{log}\left(\lambda_{i1}/\lambda_{i2}\right)$ is the log relative risk. This model is utilized to allow for heterogeneity across studies, as both the baseline risk (i.e., $\lambda_{i2}$) and log relative risk (i.e., $\xi_{i}$) may vary across studies and is a common choice for random-effects meta-analyses of a treatment effect (e.g., [7,26]).

Under the null hypothesis of no treatment effect (i.e., $\xi_{i}=0$), a sufficient statistic for the nuisance parameter, $\lambda_{i2}$, is the total number of events in the ith study, denoted as $Y_{i\cdot }=Y_{i1}+Y_{i2}$. The dot notation indicates the summation is taken over the index for the arm. We take the classical approach of basing inference on the conditional distribution of $Y_{i1}$ given the sufficient statistic, $Y_{i\cdot }$, to eliminate the nuisance parameter $\lambda_{i2}$ [7]. Simple calculations show that

(1) $$Y_{i1}\left|Y_{i\cdot }\sim \text{Binomial}\left\{Y_{i\cdot ,}\frac{1}{1+\text{exp}\left[-\left(\xi_{i}+S_{i}\right)\right]}\right\}\right.$$

where $S_{i}=\text{log}\left(N_{i1}/N_{i2}\right)$ and expit $(x)=1/\left(1+e^{-x}\right)$. As we are primarily interested in the rare event rate setting, it is important to note that basing inference on (1) justifies the exclusion of DZ studies from analysis as they do not provide information on the relative risk, $\text{exp}\left(\xi_{i}\right)$. This enables us to utilize the $K\leq K_{\text{tot}}$ non-DZ studies for analysis [27].

### 2.2. Parameter of Interest

To assess the relative event rates in the two treatment arms, we define a treatment contrast

$$\pi_{i}\equiv \text{expit}\left(\xi_{i}\right)=\frac{\lambda_{i1}}{\lambda_{i1}+\lambda_{i2}}$$

which quantifies the magnitude of the event rate in the treated group relative to the cumulative event rate across both treatment arms in the ith study. The setting of no treatment effect corresponds to $\pi_{i}=0.5$, while $\pi_{i}>0.5$ indicates the event is more common in the ith study’s treated arm relative to the control arm (e.g., the treatment has a harmful effect when the events of interest are adverse). We further assume that $\pi_{i}$ is a random effect with

$$\pi_{i}\sim \text{Beta}\left(\alpha_{0},\beta_{0}\right)\;\text{with}\;\alpha_{0},\beta_{0}>1$$

to account for between-study heterogeneity. The 0 subscript is used to denote the true values of the model parameters. We require $\alpha_{0},\beta_{0}>1$ to ensure that the random-effects distribution is unimodal so that the location parameter is identifiable.

Under the Beta random effects distribution for $\pi_{i}$, it follows that

$$\text{E}\left(\pi_{i}\right)=\frac{\alpha_{0}}{\alpha_{0}+\beta_{0}}=\mu_{0}\;\text{and}\;\text{Var}\left(\pi_{i}\right)=\mu_{0}\left(1-\mu_{0}\right)\tau_{0}=v_{0}$$

where $\tau_{0}={\left(\alpha_{0}+\beta_{0}+1\right)}^{-1}$ quantifies the between-study variability. Under the balanced design with equal sample sizes in both arms, the corresponding random effects model for $Y_{i1}|Y_{i\cdot }$ simplifies to the familiar Beta Binomial (BB) model. In the subsequent sections, we develop an exact method to make inference on $\mu_{0}$. We reparameterize the distribution of $\pi_{i}$ with respect to $\mu_{0}$ and $v_{0}$ for clarity of presentation with the following equalities:

$$\alpha_{0}=\mu_{0}\left\{\frac{\mu_{0}\left(1-\mu_{0}\right)-v_{0}}{v_{0}}\right\}\;\text{and}\;\beta_{0}=\left(1-\mu_{0}\right)\left\{\frac{\mu_{0}\left(1-\mu_{0}\right)-v_{0}}{v_{0}}\right\}.$$

Before introducing our proposal, we note that an alternative approach is to base inference on $E\left(\xi_{i}\right)$ with $\xi_{i}$ following a normal distribution. However, it is challenging to appropriately specify the standard deviation in the setting of rare events [28]. To clarify this point, consider the following toy example. Suppose we observe data from six studies with a balanced design with study-specific sample sizes of 100 and

$$\left\{\left(Y_{i1},Y_{i2}\right)=(0,20)|i=1,\ldots ,6\right\}$$

Intuition suggests that the treatment is protective. However, by placing a normal random effects distribution on the log relative risk, one cannot rule out the possibility that $\xi_{i}\sim N(10,1000)$. Under this model, there is approximately a $2^{-6}=1.5\%$ probability of obtaining the observed data by chance, which is surprisingly large given the intuition that $E\left(\xi_{i}\right)$ should be very negative. Practically, this example illustrates that it is unclear how to naturally constrain the standard deviation of the normal distribution to prevent this phenomenon from occurring.

In contrast to basing inference on $E\left(\xi_{i}\right)$, our choice of random effects distribution affords several benefits [29]. Our requirement on the support of $\left(\alpha_{0},\beta_{0}\right)$, together with the reparameterization in Section 2.2, implies that

$$v_{0}\leq \mu_{0}\left(1-\mu_{0}\right)\;\text{min}\left(\frac{\mu_{0}}{1+\mu_{0}},\frac{1-\mu_{0}}{2-\mu_{0}}\right)=v_{\text{sup}}\left(\mu_{0}\right).$$

**Figure 1.**
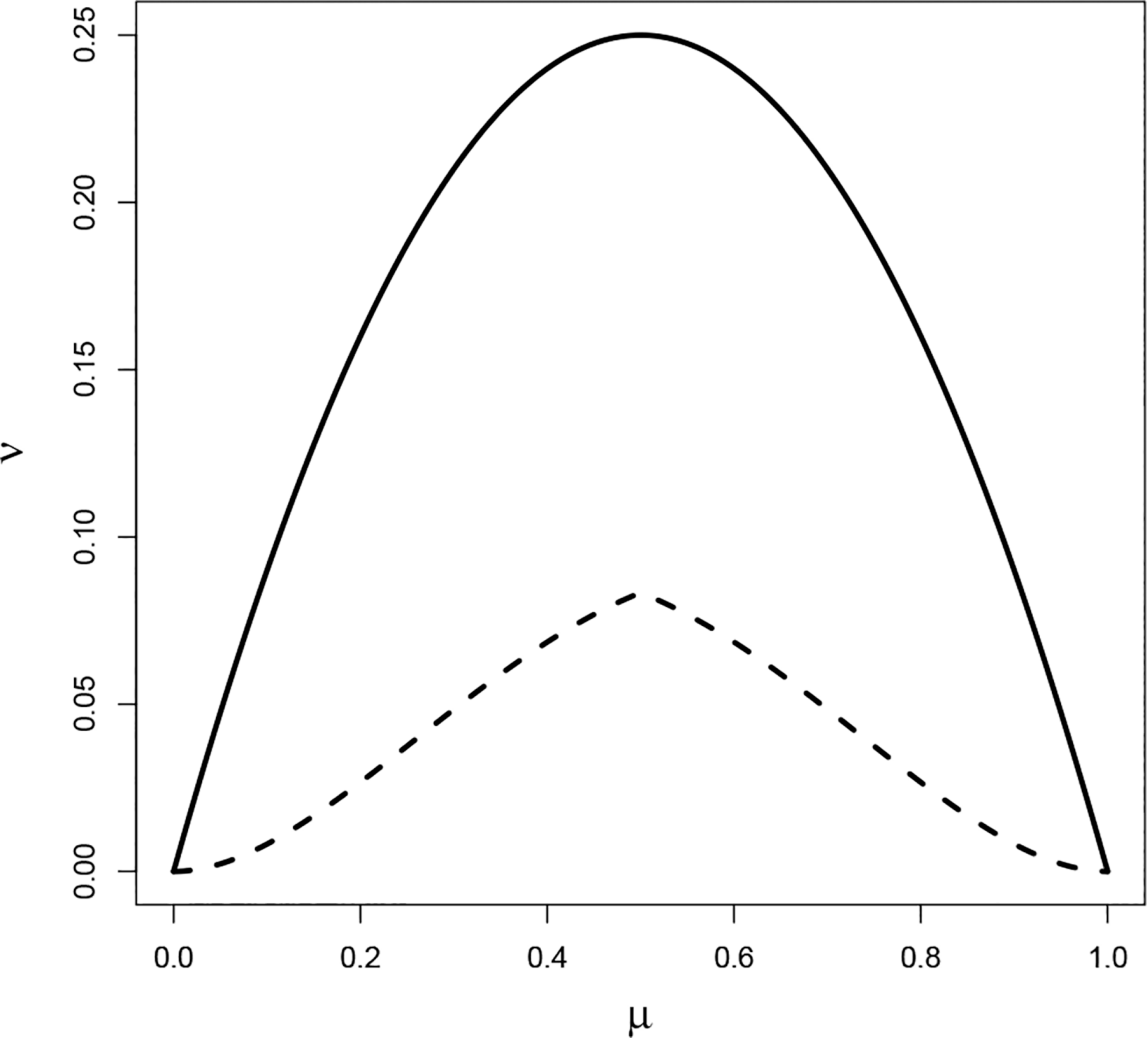
The boundary of the parameter space in the (μ,v) plane for the standard beta-binomial model (solid) and our restricted model (dashed). This image is from [29].

This constraint reduces the parameter space of the standard beta distribution defined with $\left(\alpha_{0},\beta_{0}\right)>0$ to $\left\{\left(\mu_{0},v_{0}\right)|v_{0}\leq v_{\text{sup}}\left(\mu_{0}\right)\right\}$. This assumption guarantees that the random effects distribution is unimodal so that we may (i) appropriately interpret $\mu_{0}$ as the center of $\pi_{i}$ and (ii) ensure that $\mu_{0}$ is identifiable. The parameter space is depicted in Figure 1. Going back to our toy example, one would expect that $\mu_{0}$ is close to zero. However, with $\pi_{i}\sim \text{Beta}(0.001,0.004)$, the corresponding $\mu_{0}=1/5$. Under this random effects distribution, there is approximately a 26% probability that all the $\pi_{i}\approx 0$ by chance. This paradox arises from the fact that the Beta(0.001, 0.004) distribution places 4/5 and 1/5 probability on $\pi_{i}=0$ and $\pi_{i}=1$, respectively. Our constraint naturally eliminates bimodal prior distributions to rectify this behavior.

## 3. Methods

### 3.1. Overview of the Exact Inference Procedure

While basing inference on the distribution of $Y_{i1}|Y_{i\cdot }$ eliminates the nuisance parameter dictating the event rate in the control arm, making inference on $\mu_{0}$ demands consideration of $v_{0}$. To develop an exact CI, we propose to invert unconditional hypothesis tests with respect to $v_{0}$. More specifically, we perform a test of the null hypothesis

$$H_{0}:\mu_{0}=\mu$$

based on a test statistic $T\left(\mu ;{𝒟}^{0}\right)$ that is a function of both μ and the observed data ${𝒟}^{0}$. We detail the choice of $T\left(\mu ;{𝒟}^{0}\right)$ in Section 3, and here we only assume that larger values of the test statistic lead to a rejection of $H_{0}$. The p-value function for this test is therefore defined as

$$p\left(\mu ,v;{𝒟}^{0}\right)=P\left\{T\left(\mu ;{𝒟}^{\mu ,v}\right)\geq T\left(\mu ;{𝒟}^{0}\right)|{𝒟}^{0}\right\}$$

where the probability is taken with respect to data, ${𝒟}^{\mu ,v}$, following the random effects model with parameters μ and ν outlined in Section 2.1. The unconditional test eliminates $v_{0}$ with the profile p-value (i.e., the p-value for μ that incorporates uncertainty from v) defined as

(2) $$p\left(\mu ;{𝒟}^{0}\right)=\underset{v}{\text{sup}}\;p\left(\mu ,v;{𝒟}^{0}\right).$$

**Figure 2.**
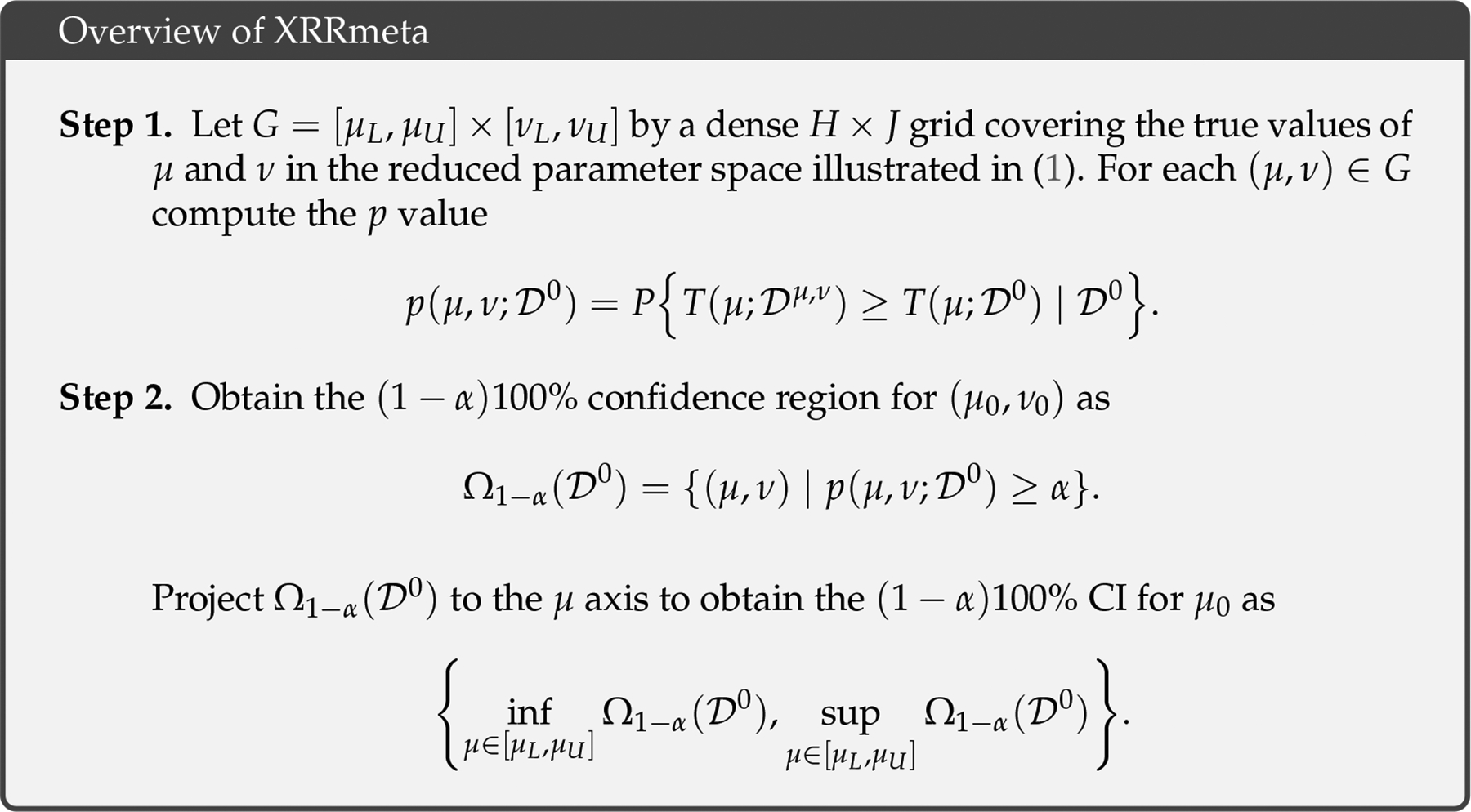
An overview of the XRRmeta procedure to produce an exact confidence interval (CI) for a random-effects meta-analysis in the setting of small, sparse settings. XRRmeta consists of two steps. The first step involves calculating p-values across different values of the parameters of the random effects model. The second step inverts the hypothesis tests from Step 1. This is done by finding all values of (μ,v) that lead to a failure to reject the null hypothesis and then projecting to the μ axis to obtain the final CI for $\mu_{0}$.

For an α-level test, the null hypothesis is rejected when $p\left(\mu ;{𝒟}^{0}\right)<\alpha$, and the corresponding (1-α)100% CI includes all μ such that $p\left(\mu ;{𝒟}^{0}\right)\geq \alpha$. Our proposed procedure, XRRmeta, utilizes this framework to yield an exact CI as detailed in Figure 2.

The primary complication in implementing XRRmeta is computing the p-value. Specifically, the cumulative distribution function of $T\left(\mu ;{𝒟}^{\mu ,v}\right)$ is required to calculate $p\left(\mu ,v;{𝒟}^{0}\right)$. As the distribution function is likely unavailable in analytic form, an approximation can be obtained with Monte Carlo (MC) simulation. More specifically, for a large number M, we can repeat the following steps for m=1,…,M within Step 1 of the XRRmeta method in Figure 2:

**Step 1a.** Generate a simulated dataset, ${𝒟}_{m}=\left\{\left(Y_{i1}^{m},Y_{i2}^{m}\right)|i=1,\ldots ,K\right\}$ where $Y_{i2}^{m}=Y_{i\cdot }-Y_{i1}^{m}$,
$$Y_{i1}^{m}\sim \text{Binom}\left[\text{expit}\left\{\text{logit}\left(\pi_{i}\right)+S_{i}\right\}\right],$$
  $\pi_{i}\sim \text{Beta}(\mu ,v)$, and $S_{i}=\text{log}\left(N_{i1}/N_{i2}\right)$.
**Step 1b.** Compute the test statistic $T_{m}=T\left(\mu ;{𝒟}_{m}\right)$.

Using Steps 1a and 1b, the p-value, $p\left(\mu ,v;{𝒟}^{0}\right)$, is computed with its empirical counterpart from the MC replications, $M^{-1}\sum_{m=1}^{M}I\left\{T_{m}\geq T\left(\mu ;{𝒟}^{0}\right)\right\}$, which is the proportion of simulated test statistics that are as or more extreme than the observed value. However, the MC procedure must be executed HJ times, which is computationally burdensome with a large value of M. We therefore strategically design $T\left(\mu ;{𝒟}^{0}\right)$ to allow for fast computation of the exact CI.

### 3.2. Proposed Test Statistic for the Exact Inference Procedure

To motivate our proposal, we begin by introducing the test statistic under a balanced design in which the underlying random effects model reduces to the BB model with parameters $\mu_{0}$ and $v_{0}$. We then describe an augmentation of the proposed test statistic under the more likely scenario of an unbalanced design and further simplifications to accelerate computation.

#### 3.2.1. Balanced Design

We first consider the setting when all studies included in the meta-analysis utilize a balanced design. A balanced design occurs when participants in each trial are assigned to the treated and control arms at the same rate, which can help to improve statistical power in detecting a treatment effect. Under the balanced design, $N_{i1}=N_{i2}$ for i=1,…,K so that $Y_{i1}|Y_{i\cdot }\sim \text{BB}\left(Y_{i\cdot },\mu_{0},v_{0}\right)$. Natural choices of $T\left(\mu ;{𝒟}^{0}\right)$ include the Wald, score, or likelihood ratio test statistics. We propose a Wald statistic using method of moments estimators for $\mu_{0}$ and $v_{0}$, as they do not require iterative calculations like maximum likelihood estimators that would substantially increase the computational time of the MC procedure of XRRmeta. To this end, note that under the balanced design

(3) $$\text{E}\left(\frac{Y_{i1}}{Y_{i\cdot }}\right)=\mu_{0}\;\text{and}\;\text{E}\left\{{\left(\frac{Y_{i1}}{Y_{i\cdot }}\right)}^{2}\right\}=\left(1-\frac{1}{Y_{i\cdot }}\right)\left(\mu_{0}^{2}+v_{0}\right)+\frac{\mu_{0}}{Y_{i\cdot }}.$$

We obtain the method of moments estimator for the true model parameter, $\mu_{0}$, as

$$\hat{\mu }=K^{-1}\sum_{i=1}^{K}\frac{Y_{i1}}{Y_{i\cdot }}.$$

Letting ${\tilde{Y}}_{i1}=Y_{i1}+0.5$, ${\tilde{Y}}_{i\cdot }=Y_{i\cdot }+1$, and ${\hat{\mu }}_{int}=K^{-1}\sum_{i=1}^{K}\frac{{\tilde{Y}}_{i1}}{{\tilde{Y}}_{i\cdot }}$, we obtain the method of moments estimator for $v_{0}$ as

(4) $$\hat{v}=\text{max}\left\{0,\frac{\sum_{i=1}^{K}\left\{{\left(\frac{{\tilde{Y}}_{i1}}{{\tilde{Y}}_{i\cdot }}\right)}^{2}-\frac{{\hat{\mu }}_{int}}{{\tilde{Y}}_{i\cdot }}\right\}}{\sum_{i=1}^{K}\left(1-\frac{1}{{\tilde{Y}}_{i\cdot }}\right)}-{\hat{\mu }}_{int}^{2}\right\}.$$

The continuity correction in (4) is utilized for estimating $\nu_{0}$ so that (i) $\text{Var}\left(\left.\frac{{\tilde{Y}}_{i1}}{{\tilde{Y}}_{i}}\right|\pi_{i}\right)>0$ when $Y_{i1}=0$ or $Y_{i1}=Y_{i\cdot }$ in all K studies and (ii) all studies contribute to estimation as it is possible that $Y_{i\cdot }=1$ in the rare event setting [6]. In contrast to existing procedures, this correction does not impact the validity of XRRmeta, as it is based on the exact distribution of the test statistic. We select 0.5 as a historical convention and because it is a small number to minimize bias [7]. The test statistic for the familiar Wald test of the null hypothesis $H_{0}:\mu_{0}=\mu$ is simply

$$T\left(\mu ;{𝒟}^{0}\right)=\frac{(\hat{\mu }-\mu {)}^{2}}{\hat{\text{Var}}(\hat{\mu })}$$

where the estimated variance is given by $\hat{\text{Var}}(\hat{\mu })=K^{-2}\left\{\sum_{i=1}^{K}\frac{\hat{\mu }(1-\hat{\mu })}{{\hat{Y}}_{i\cdot }}+\left(1-\frac{1}{{\hat{Y}}_{i}}\right)\hat{v}\right\}$.

It is important to note that the choice of test statistic for XRRmeta is not unique. In addition to computational efficiency, it is necessary to consider the impact of the test statistic on statistical efficiency and hence the length of the resulting CI. For example, one may also estimate $\mu_{0}$ with $\sum_{i=1}^{K}Y_{i1}/\sum_{i=1}^{K}Y_{i}$, which is expected to be more accurate in the presence of low between-study heterogeneity. Additionally, while the inverse variance estimator (i.e., weighting each study inversely to its variance for point estimation) is generally expected to provide the best performance, this is true only when the total number of events of every individual study is sufficiently large. We initially investigated the inverse variance estimator of $\mu_{0}$ in our numerical studies, but did not observe a substantial improvement in the efficiency of XRRmeta relative to our proposed method of moments estimators.

#### 3.2.2. Unbalanced Design

Building on Section 3.2.1, we next consider the more realistic setting of an unbalanced design in which the treated and control arms have different sample sizes for at least one study. In this setting, $Y_{i1}|Y_{i\cdot }$ no longer follows the familiar BB model. The first two moments are $\text{E}\left(\frac{Y_{i1}}{Y_{i}}\right)=\text{E}\left\{\text{expit}\left(\xi_{i}+S_{i}\right)\right\}$ and

$$\text{E}\left\{{\left(\frac{Y_{i1}}{Y_{i\cdot }}\right)}^{2}\right\}=\text{E}\left[\frac{\text{expit}\left(\xi_{i}+S_{i}\right)}{Y_{i\cdot }}+\left(1-\frac{1}{Y_{i\cdot }}\right){\left\{\text{expit}\left(\xi_{i}+S_{i}\right)\right\}}^{2}\right].$$

The previously proposed method of moment estimators from the balanced design setting therefore cannot be directly calculated in the unbalanced design setting. To avoid estimators requiring iterative calculation (e.g., maximum likelihood estimators), we propose weighted counterparts of our previous proposals motivated by “resampling” a subset of the larger arm to mimic the balanced design setting. For each study, the possible outcomes for the event of interest are enumerated under a balanced design and assigned a weight according to their likelihood conditional on the observed data. The moment estimators are then calculated with the resampled data and corresponding weights. The resampling procedure enables us to use weighted method of moment estimators to maintain a computationally efficient procedure for XRRmeta.

For illustration, we detail the case with $N_{i1}>N_{i2}$ for i=1,…,K. Analogous results hold for the setting with smaller treatment groups and thus for any combination of imbalance in treatment and control arm sample sizes across the studies. Given the observed data, the possible outcomes for the ith study under a balanced design can be enumerated as

$${𝒟}_{i}^{*}=\left\{\left(Y_{i1l}^{*},N_{i2}\right),\left(Y_{i2},N_{i2}\right)|l=\text{max}\left(0,N_{i2}-N_{i1}+Y_{i1}\right),\ldots ,Y_{i1}\right\}$$

where $Y_{i1l}^{*}=l$. The probability of observing l events in the treated arm is dictated by the hypergeometric distribution defined as

pi1l=Ni1-Yi1Ni2-lYi1l(Ni1Ni2).

More concretely, suppose the data for two studies are

$${𝒟}_{1}^{O}=\left\{\left(Y_{11},N_{11}\right),\left(Y_{12},N_{12}\right)\right\}=\left\{\left(2,80\right),\left(1,50\right)\right\}\;{𝒟}_{2}^{O}=\left\{\left(Y_{21},N_{21}\right),\left(Y_{22},N_{22}\right)\right\}=\left\{\left(1,100\right),\left(0,90\right)\right\}$$

For the first study, the possible outcomes under the balanced design are given by

$${𝒟}_{1}^{*}=\left\{\left(Y_{11l}^{*},N_{12}\right),\left(Y_{12},N_{12}\right)|l=0,\ldots Y_{11}\right\}\;=\{[(0,50),(1,50)],[(1,50),(1,50)],[(2,50),(1,50)]\}.$$

Here, we have sampled fifty of the eighty patients in the first study’s treatment arm, fifty being the size of the smaller arm, of which up to two experience events. For the second study, we similarly obtain ${𝒟}_{2}^{*}=\{[(0,90),(0,90)],[(1,90),(0,90)]\}$. In this study, and more generally when there is a study with zero events in the control arm, we obtain a DZ study. As our approach removes DZ studies, we employ the following correction to proceed with moment estimation as in Section 3.2.1:

$${𝒟}_{i}^{*c}=\left\{\left(Y_{i1l}^{*},N_{i1l}\right),\left(Y_{i2},N_{i2}\right)|l\in ℒ\right\}\;\text{where}\;ℒ=\left\{\begin{array}{ll} 0,\ldots ,Y_{i1} & \text{if}\;Y_{i2}\neq 0\\ 1,\ldots ,Y_{i1} & \text{if}\;Y_{i2}=0 \end{array}\right.$$

and $p_{i1l}^{c}=p_{i1l}/\sum_{l\in ℒ}p_{i1l}$. Letting $Y_{i\cdot l}^{*}=Y_{i1l}^{*}+Y_{i2}$, we then estimate $\mu_{0}$ based on ${𝒟}_{i}^{*c}$ and $p_{i1l}^{c}$ as

$$\tilde{\mu }=K^{-1}\sum_{i=1}^{K}\sum_{l\in ℒ}\frac{Y_{i1l}^{*}}{Y_{i\cdot l}^{*}}p_{i1l\cdot }^{c}$$

With ${\tilde{Y}}_{i1l}^{*}=Y_{i1l}^{*}+0.5,{\tilde{Y}}_{i\cdot l}^{*}=Y_{i\cdot l}^{*}+1$, and

$${\tilde{\mu }}_{int}=K^{-1}\sum_{i=1}^{K}\sum_{l\in ℒ}\frac{{\tilde{Y}}_{i1l}^{*}}{{\tilde{Y}}_{i\cdot l}^{*}}p_{i1l^{,}}^{c}$$

we estimate $v_{0}$ as

$$\tilde{v}=\text{max}\left\{0,\frac{\sum_{i=1}^{K}\sum_{l\in ℒ}\left\{{\left(\frac{{\tilde{Y}}_{i1l}^{*}}{{\tilde{Y}}_{i\cdot l}^{*}}\right)}^{2}-\frac{{\tilde{\mu }}_{int}}{{\tilde{Y}}_{i\cdot l^{*}}}\right\}p_{i1l}^{c}}{\sum_{i=1}^{K}\sum_{l\in ℒ}\left(1-\frac{1}{{\tilde{Y}}_{i\cdot l}^{*}}\right)p_{i1l}^{c}}-{\tilde{\mu }}_{int}^{2}\right\}.$$

The test statistic is taken as

$$T\left(\mu ;{𝒟}^{0}\right)=\frac{(\tilde{\mu }-\mu {)}^{2}}{\hat{\text{Var}}(\tilde{\mu })}$$

where $\hat{\text{Var}}(\tilde{\mu })=K^{-2}\left\{\sum_{i=1}^{K}\sum_{l\in ℒ}p_{i1l}^{c}\frac{\tilde{\mu }(1-\tilde{\mu })}{{\tilde{Y}}_{i\cdot l}^{*}}+p_{i1l}^{c}\left(1-\frac{1}{{\tilde{Y}}_{i\cdot l}^{*}}\right)\tilde{v}\right\}$.

Though not straightforward to verify analytically, our numerical studies coincide with the intuition that our proposed estimator is consistent for the first and second moments in settings with sufficiently high event rates in the control arm. Our adjustment for DZ studies biases the point estimator away from the null with the magnitude of bias depending on the degree of imbalance and the rarity of the event. The validity of XRRmeta still holds, however, as inference is based on the exact distribution of $T\left(\mu ;{𝒟}^{0}\right)$. The bias only affects efficiency and therefore the width of the resulting CI. Our numerical studies indicate that our proposed test statistic does not result in overly conservative inference, suggesting that efficiency is not greatly affected.

### 3.3. Computational Details

Another useful feature of our proposed test statistic is that it enables us to significantly accelerate the computation time of XRRmeta. Specifically, we can reduce the computational complexity of XRRmeta from the O(HJ) operations detailed in Figure 2 to O(H) operations using a stochastic dominance result for the test statistic. Specifically, $T\left(\mu ;{𝒟}^{\mu ,v_{1}}\right)$ appears to generally first-order stochastically dominate $T\left(\mu ;{𝒟}^{\mu ,v_{2}}\right)$ for a fixed μ and $v_{1}<v_{2}$ [29]. The numerical studies in the current manuscript as well as in our prior work suggest that

$$P\left\{T\left(\mu ;{𝒟}^{\mu ,v_{1}}\right)>t\right\}<P\left\{T\left(\mu ;{𝒟}^{\mu ,v_{2}}\right)>t\right\}$$

for t in the tail region of interest [29]. Intuitively, this property follows from the fact that a random effects distribution with higher variability will yield a test statistic with more variation and hence a larger tail probability. Practically, this property may be leveraged to significantly decrease execution time, as it implies the profile p value for μ can be obtained by simply calculating the p value at the largest value of v in the parameter space. That is,

$$p\left(\mu ;{𝒟}^{0}\right)=\underset{v}{\text{sup}}\;p\left(\mu ,v;{𝒟}^{0}\right)\approx p\left\{\mu ,v_{\text{sup}}(\mu );{𝒟}^{0}\right\}.$$

The computational complexity of XRRmeta is thus reduced to O(H) operations by computing $p\left(\mu ;{𝒟}^{0}\right)$ with the values along the boundary of the parameter space.

Supplementary Section S2 details the complete execution of XRRmeta. The procedure involves three steps: (1) initialization, (2) iteration, and (3) correction. The initialization step is used to further accelerate computation by beginning the grid search using asymptotic confidence bounds whenever possible. The iteration step leverages the stochastic dominance result to move along the boundary of the parameter space to determine initial upper and lower limits of the CI. The correction step checks values of (μ,v) beyond the CI limits found in the iteration step, as the stochastic dominance result is only an approximation. In terms of the implementation of XRRmeta, a key decision is the choice of grid size, s, used to iterate along the boundary of the parameter space. We suggest using the original scale to explicitly control the precision for making inferences on the treatment contrast. Taking s=0.001 is reasonable for the magnitude of most effect sizes and was utilized in our simulation and real data analyses. The number of MC iterations similarly depends on the desired precision. A reasonable choice is M of at least 2000, as evaluating a p-value of 0.05 with a standard error of 0.005 requires M≥2000 [29]. The number of (μ,v) pairs to evaluate in the correction step can simply be taken as a multiple of s. We found that 10s was sufficient in our numerical studies.

## 4. Results

We next evaluate the performance of XRRmeta in both simulated and real data. An example analysis with our R package can be found in Supplementary Section S5.

### 4.1. Simulation Studies

We evaluated the performance of XRRmeta through extensive simulation studies. In all settings, the observed data were generated as

$$Y_{ij}\sim \text{Poisson}\left(N_{ij}\lambda_{ij}\right)$$

where $\lambda_{i1}\sim \text{Gamma}\left(\alpha_{0},\alpha_{0}/r_{0}\right)$ and $\lambda_{i2}\sim \text{Gamma}\left(\beta_{0},\alpha_{0}/r_{0}\right)$. We used this data generating mechanism to achieve an average event rate of $r_{0}$ in the treated arm and an average event rate of $r_{0}\beta_{0}/\alpha_{0}$ in the control arm. The scale parameters of the gamma distribution for both arms are identical so that $\pi_{i}\sim \text{Beta}\left(\alpha_{0},\beta_{0}\right)$. We varied (i) the total number of studies, $K_{\text{tot}}$, (ii) the event rate in the treated arm, $r_{0}$, and (iii) the values of $\alpha_{0}$ and $\beta_{0}$ to evaluate XRRmeta across a wide range of scenarios. Varying $K_{\text{tot}}$ allows us to assess the impact of the dataset size on the performance of our procedure while varying $r_{0}$ allows us to assess the impact on the event rate. Varying $\alpha_{0}$ and $\beta_{0}$ represents varying degrees of treatment effect and between-study heterogeneity. The three primary settings, representing high, moderate, and low between-study heterogeneity, are summarized in Table 1.

In the settings with a protective effect, the values of $\left(\alpha_{0},\beta_{0}\right)$ were selected to achieve a relative risk of 0.67 $\left(\mu_{0}=0.4\right)$ and to maintain a similar between-study heterogeneity to the setting of no treatment effect. The between-study variation for Settings 1, 2, and 3 is approximately $v_{0}=0.064,0.021$, and 0.001, respectively. In all settings, we considered $r_{0}=0.01$ and 0.03 with the number of studies K=12,24,48, and 96. The percentage of DZ studies was approximately 15% on average across the settings with $r_{0}=0.01$ and near 0% with $r_{0}=0.03$. The study-specific sample sizes $\left(N_{i1},N_{i2}\right)$ were randomly sampled from the rosiglitazone study reported in Supplementary Table S1. All results were averaged over 2000 replications.

To assess the conservativeness and efficiency of XRRmeta while facilitating comparisons across the most commonly used existing meta-analytic methods for the odds ratio, we summarized the Type I error for the null effect settings and the power for the protective effect settings. As the odds ratio and relative risk are comparable in the rare events setting, we only report results for the former. We compared XRRmeta to five common methods, including the Mantel–Haenszel method with and without a 0.5 continuity correction for zero event studies $\left(\text{MH},{\text{MH}}_{\text{cc}}\right)$, the fixed and random effects Peto method (Peto-F, Peto-R), and the DerSimonian–Laird method with a 0.5 continuity correction for zero event studies (DL). These methods were implemented with the *metabin* package in R.

**Figure 3.**
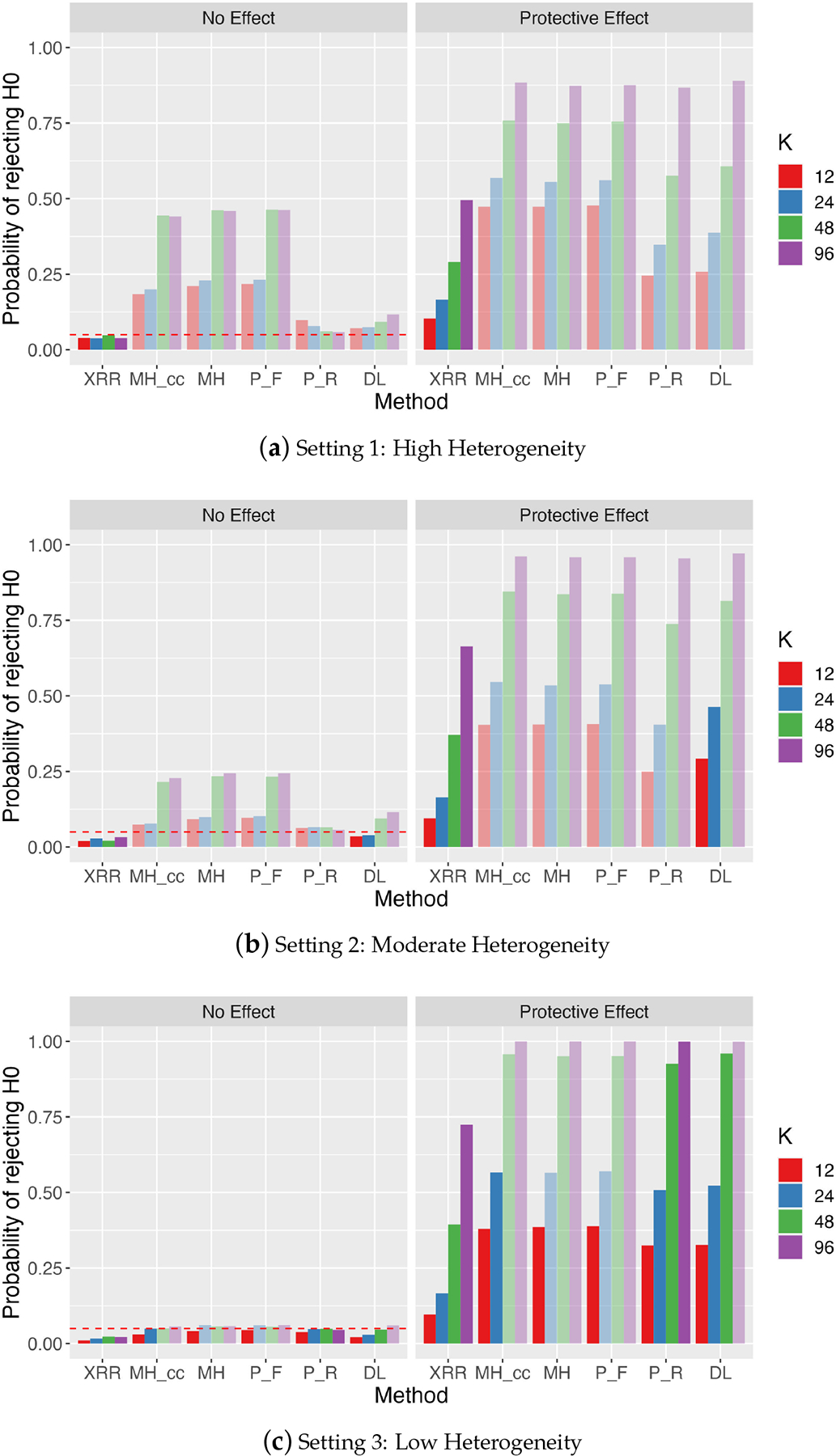
Type I error and power with $r_{0}=0.01$ for XRRmeta (XRR), Mantel–Haenszel with and without a 0.5 continuity correction $\left(\text{MH},{\text{MH}}_{cc}\right)$, the fixed and random effects Peto method (P_F, P_R), and the DerSimonian–Laird method with a 0.5 continuity correction (DL). Methods that do not control the Type I error are shown in a lighter shade.

Type I error and power for all three settings with $r_{0}=0.01$ are summarized in Figure 3. Methods with inflated Type I error (i.e., Type I error exceeding the nominal level of 0.05) are presented in a lighter shade as the power may not be properly interpreted [30]. Overall, XRRmeta is the only method that consistently controls Type I error across sample sizes and heterogeneity levels, which is expected as XRRmeta is an exact inference procedure. The setting of high between-study heterogeneity is particularly striking as none of the comparators provides a valid test of the treatment difference at any of the study sizes considered. Moreover, all of the comparators, with the exception of the random effects Peto method, exhibit the counterintuitive property that the Type I error becomes more inflated as the number of studies meta-analyzed increases, precisely the setting where practitioners would expect the conclusions to become more reliable.

The fixed effect approaches (Mantel–Haenszel with or without continuity correction and Peto fixed effect) have substantially inflated Type I error in the high and moderate heterogeneity settings. The random effects comparators (Peto random effects and DerSimonian–Laird) better cope with between-study heterogeneity, but still do not maintain the Type I error. Similar to the fixed effect approaches, the DL method exhibited Type I error increasing with the number of studies, a phenomenon that has been reported previously [7]. Lastly, we also examined the empirical coverage level of the constructed CIs across different settings. Note that one minus the Type I errors reported in Figure 3 are the observed coverage levels of CIs under the null. The empirical coverage levels are always above 95%, as the proposed exact procedures ensured. In addition, the coverage levels in most settings are below 98%, suggesting that the resulting exact intervals are not overly conservative.

The results for $r_{0}=0.03$ are presented in Supplementary Figure S1. The same patterns are observed, but higher power is achieved for all methods due to the higher event in the treated group. Together, our results demonstrate the utility of fixed effect methods when between-study homogeneity is expected and the benefit of XRRmeta in uniformly controlling Type I error when between-study heterogeneity is expected.

### 4.2. Real Data Examples

We next evaluate the performance of the proposed procedure in two real data examples. The first is our motivating example, the rosiglitazone study, where the proportion of DZ studies is high as the events of interest are extremely rare. The second involves a recent meta-analysis of the utility of face masks in preventing person-to-person transmission of SARS-CoV-2 and COVID-19, which has fewer studies but a relatively higher event rate compared with the rosiglitazone study. We selected the COVID-19 study due to the controversy over mask use during the pandemic and to evaluate XRRmeta in the small data setting. For both analyses, we present the results from XRRmeta and the five comparison methods utilized in the simulation studies ($\text{MH},{\text{MH}}_{\text{cc}}$, Peto-F, Peto-R, DL) for consistency. Some of these methods were used in the initial analyses of the two datasets.

#### 4.2.1. Rosiglitazone Study

Rosiglitazone is a popular drug for the treatment of Type II diabetes mellitus. Its effect on cardiovascular mortality (CVD) and myocardial infarction (MI) has been under scrutiny since the 2007 publication of Niessen and Wolski’s meta-analysis of 42 randomized controlled clinical trials in the *New England Journal of Medicine* [9,10]. The data depicted in Supplementary Table S1 include the forty-two studies reported in the original paper as well as six additional DZ studies that were excluded from analysis. In the original analysis, the authors utilized the fixed-effect Peto method. They found that in the rosiglitazone group, as compared with the control group, the odds ratio for MI was 1.43 with a 95% CI of [1.03, 1.98], while the odds ratio for CVD was 1.64 with a 95% CI of [0.98, 2.74]. However, these conclusions are questionable for numerous reasons, including the exclusion of DZ trials and the use of a fixed-effect approach. For instance, including the DZ studies and employing the Mantel–Haenszel method with a continuity correction of 0.5 in all studies results in a contradictory conclusion for the MI endpoint (Table 2). Tian et al. obtained similar results in their re-analysis of the data using their proposed exact confidence procedure for the risk difference under a fixed-effect model [13]. However, a conventional fixed-effect approach is not appropriate for the rosiglitazone data, as two very large studies are present in the data analysis [16]. For example, excluding the two larger studies from Niessen and Wolski’s analysis yields substantially different intervals of [0.88, 2.39] and [1.17, 4.91] for MI and CVD, respectively. A random effects approach that weighs all studies equally is more appropriate for the rosiglitazone data.

We report the results for the treatment contrast from XRRmeta and the comparison methods for the odds ratio in Table 2. In terms of the comparison methods, the CIs from the fixed effect Peto method correspond to the original analysis for both outcomes because the six additional DZ studies do not influence the results. For the MI outcome, the random effects Peto CI is identical to the fixed effect model. Both the Peto CIs and the Mantel–Haenszel CI without continuity correction coincide with the original conclusions of a significant effect. Adding a continuity correction to the Mantel–Haenszel analysis or utilizing the Dersimonian–Laird method reverses the conclusion. For CVD, these two methods demonstrate that there is no statistically significant evidence of increased risk of an adverse event with rosiglitazone, while the Mantel–Haenszel method without continuity correction and both Peto methods show marginal significance of a positive effect.

For XRRmeta, both exact CIs exclude the null value of 0.5. More specifically, our results are consistent with the original study for the MI endpoint, indicating that there is evidence for an increased risk of MI among patients receiving rosiglitazone. However, we obtain conflicting results for CVD. Our analysis suggests the frequency of CVD in the treated arm is significantly higher than in the control arm, which is consistent with the random effects analysis of Shuster et al. [16]. Unlike the five comparison methods, we reiterate that our procedure is justified in its exclusion of DZ studies as it is based on a conditional inference argument. Additionally, XRRmeta is guaranteed to provide valid inference in settings with a relatively small number of studies and between-study variability. The difference in results across the methods could be due to the large number of DZ studies, the presence of the two larger studies, and/or between-study heterogeneity in the CVD analysis, issues that can impact the validity of the comparison methods, but not XRRmeta. Additionally, the conflicting conclusions between methods are consistent with our simulation findings.

#### 4.2.2. Face Mask Study

COVID-19 is caused by severe acute respiratory syndrome coronavirus 2 (SARS-CoV-2) and is transmitted from person to person through close contact. In the midst of the COVID-19 pandemic, Chu et al. conducted a systematic review to evaluate the preventative effect of face masks on virus transmission in healthcare and community settings [31]. At this time, SARS-CoV-2 had infected nearly 6 million individuals worldwide and caused more than 359,000 deaths. Vaccinations were not widespread, and reducing the infection rate was a global priority. The authors aimed to provide evidence for the use of face masks in preventing person-to-person virus transmission as their use was heavily debated by media and public health authorities. Twenty-nine studies met the inclusion criteria for their study and the resulting data are presented in Supplementary Table S2. The authors obtained an estimated relative risk of 0.34 with a 95% CI of [0.26, 0.45] with the DL method excluding the DZ studies, indicating substantially reduced transmission with face masks.

We report the results for the treatment contrast from XRRmeta and the comparison methods for the odds ratio in Table 3. The CIs from all five comparison methods yield similar results in terms of the magnitude of the estimated odds ratio, which indicates that face masks are effective in preventing person-to-person virus transmission. As expected, and consistent with the simulation study findings, the random effects methods (DL, Peto-R) have larger CIs than the fixed effects methods ($\text{MH},{\text{MH}}_{\text{cc}}$, Peto-F). Our proposal, XRRmeta, yields a conclusion consistent with the results of the comparison methods, as the exact CI for $\mu_{0}$ is well below 0.5. Overall, there is clear evidence of reduced person-to-person virus transmission with face mask use across all our analyses. Moreover, these results echo our simulation studies, which illustrate that XRRmeta is not substantially underpowered relative to the comparison methods we have considered, particularly in this example with few studies.

## 5. Discussion

We introduced a new method, XRRmeta, for performing a random effects meta-analysis of the treatment effect in a two-group comparison with small, sparse datasets. As noted by Zabriskie et al., the current setting has been largely underappreciated in the meta-analysis literature. The *Cochrane Handbook for Systematic Reviews of Interventions* suggests that “incorporation of heterogeneity into an estimate of a treatment effect should be a secondary consideration when attempting to produce estimates of effects from sparse data—the primary concern is to discern whether there is any signal of an effect in the data” [32]. Our simulation studies, however, illustrate that the presence of heterogeneity has a large bearing on the conclusions drawn from meta-analyses in such settings. When compared with the most commonly used meta-analysis methods, XRRmeta was the only method that uniformly maintained the Type I error without yielding overly conservative inference (Figures 3 and S1). These findings were consistent across the various settings considered in Section 4.1, with different between-study heterogeneity, event rates, and numbers of studies included in the meta-analyses. In real data applications of two very different collections of studies, we found evidence for increased incidence of myocardial infarction and cardiovascular death among diabetics treated with rosiglitazone and for reduced incidence of person-to-person COVID-19 transmission among face mask users. Additionally, we have released an R package implementing XRRmeta to encourage its use by practitioners (https://github.com/zrmacc/RareEventsMeta (accessed on 16 October 2024)).

It is important to note that the performance of our procedure inherently depends on the choice of test statistic. Our numerical studies and real data analyses indicate that the chosen Wald test statistic provides a reasonable balance between statistical efficiency and computational speed. For example, the rosiglitazone study took roughly 30 min to run (Intel Core i7-1060NG7 @ 1.2 GHz). While the comparison methods took approximately 2 min to run, the fixed approaches are not appropriate for data with heterogeneity across studies and existing random effects do not control Type I error. Importantly, knowledge of potential differences across studies can guide practitioners in the choice of a fixed effect or random effects approach. A key consideration in the use of XRRmeta relative to classical counterparts is the price paid in terms of power to control Type I error. While the XRR method favors conservatism, it is ultimately a question of the goal of the specific meta-analysis if controlling Type I error is the priority. For instance, maintaining the integrity of Type I error in drug safety studies, such as the rosiglitazone case, can prevent the unnecessary removal of a drug from the market or the imposition of restrictive safety measures that could deny patients access to a beneficial treatment. However, in certain situations, Type I error may be less critical, particularly when a conservative approach is preferred in assessing the potential harmful effects of a drug. Lastly, our future work will include improvements to XRRmeta, including the use of parallelization techniques to further expedite computation and an extension to the analysis of incidence rates. We also plan to conduct a large-scale validation study in similar spirit to Tsujimoto et al., who investigated 885 meta-analyses in Cochrane reviews, to investigate the impact of XRRmeta in favoring conservatism and to develop sample size calculations [33].

## Supplementary Material

supplementary materials

**Supplementary Materials:** The following supporting information can be downloaded at: https://www.mdpi.com/article/10.3390/stats8010005/s1, Table S1: Data for the rosiglitazone study; Figure S1: Type I error and power with ${\text{r}}_{0}=0.03$ for XRRmeta (XRR), Mantel-Haenszel with and without a 0.5 continuity correction (MH, MH-CC), the fixed and random effects Peto method (Peto-F, Peto-R), and the DerSimonian-Laird method with a 0.5 continuity correction (DL); Table S2: Data for the face mask study.

## Figures and Tables

**Table 1.** Parameters of the Beta distribution for the three primary simulation settings.

Setting	Treatment Effect	$\left(\alpha_{0},\beta_{0}\right)$
1: High Heterogeneity	Null	(1.45, 1.45)
	Protective	(1.10, 1.65)
2: Moderate Heterogeneity	Null	(5.50, 5.50)
	Protective	(4.20, 6.30)
3: Low Heterogeneity	Null	(145, 145)
	Protective	(110, 165)

**Table 2.** Meta-analysis results for the myocardial infarction (MI) and cardiovascular death (CVD) endpoints of the rosiglitazone study. Point estimates of the odds ratios, the 95% confidence intervals (CI), and p-values from the Mantel–Haenszel method with and without a 0.5 continuity correction $\left(\text{MH},{\text{MH}}_{\text{cc}}\right)$; the fixed and random effects Peto method (Peto-F, Peto-R); and the DerSimonian–Laird method with a 0.5 continuity correction for zero event studies (DL), as well as the treatment contrast results from XRRmeta. Statistically significant results are in bold. Note that the reference value of the treatment contrast from XRRmeta is 0.5, and increasing values (approaching 1.0) indicate a concentration of the event in the treatment group.

Endpoint	Method	Point Estimates	CI	CI Length	p-Value
MI	MH	1.42	[1.03, 1.98]	0.95	**0.033**
	${\text{MH}}_{\text{cc}}$	1.23	[0.92, 1.65]	0.73	0.163
	Peto-F	1.43	[1.03, 1.98]	0.95	**0.032**
	Peto-R	1.43	[1.03, 1.98]	0.95	**0.032**
	DL	1.23	[0.91, 1.67]	0.76	0.178
	XRRmeta	0.67	[0.51, 0.82]	0.31	**0.047**
CVD	MH	1.70	[0.98, 2.93]	1.95	0.057
	${\text{MH}}_{\text{cc}}$	1.13	[0.76, 1.69]	0.93	0.541
	Peto-F	1.64	[0.98, 2.74]	1.76	0.060
	Peto-R	1.64	[0.98, 2.74]	1.76	0.060
	DL	1.10	[0.73, 1.66]	0.93	0.662
	XRRmeta	0.79	[0.56, 0.90]	0.34	**0.010**

**Table 3.** Meta-analysis results for the face mask study. Point estimates of the odds ratios, the 95% confidence intervals (CI), and p-values from the Mantel–Haenszel method with and without a 0.5 continuity correction $\left(\text{MH},{\text{MH}}_{\text{cc}}\right)$; the fixed and random effects Peto method (Peto-F, Peto-R); and the DerSimonian–Laird method with a 0.5 continuity correction for zero event studies (DL), as well as the treatment contrast results from XRRmeta. Note that the reference value of the treatment contrast from XRRmeta is 0.5, and decreasing values (approaching 0.0) indicate a depletion of the event from the treatment group.

Method	Point Estimates	CI	CI Length	p-Value
MH	0.22	[0.18, 0.28]	0.10	<0.0001
${\text{MH}}_{\text{cc}}$	0.23	[0.18, 0.28]	0.10	<0.0001
Peto-F	0.27	[0.22, 0.32]	0.10	<0.0001
Peto-R	0.24	[0.18, 0.33]	0.15	<0.0001
DL	0.22	[0.16, 0.32]	0.16	<0.0001
XRRmeta	0.19	[0.11, 0.27]	0.16	<0.005

## Data Availability

The data from the Rosiglitazone Study and the Face Mask Study are included in the Supplemental Materials.
