# Supplementary material for: Exact Inference for Random Effects Meta-Analyses for Small, Sparse Data
Source: Stats (Basel). Author manuscript; Available in PMC 2025 Sep 24. (PMC12456449; doi:10.3390/stats8010005)
Supplement: supplementary materials [file NIHMS2106192-supplement-supplementary_materials.pdf]

Supplementary Materials for *Exact Inference for Random Effects Meta-Analyses with Small, Sparse Data*

Jessica Gronsbell[1], Zachary R. McCaw[2], Timothy Regis[1], Lu Tian[3]

Correspondence: j.gronsbell@utoronto.ca.

University of Toronto[1], Harvard School of Public Health[2], Stanford University[3]

1 Rosiglitazone Data

	Rosiglitazone			Control		
Study ID	N	CVD	MI	N	CVD	MI
1	357	1	2	176	0	0
2	391	0	2	207	0	1
3	774	0	1	185	0	1
4	213	0	0	109	0	1
5	232	1	1	116	0	0
6	43	0	0	47	0	1
7	121	0	1	124	0	0
8	110	3	5	114	2	2
9	382	0	1	384	0	0
10	284	0	1	135	0	0
11	294	2	0	302	1	1
12	563	0	2	142	0	0
13	278	0	2	279	1	1
14	418	0	2	212	0	0
15	395	2	2	198	0	1
16	203	1	1	106	1	1
17	104	0	1	99	0	2
18	212	1	2	107	0	0
19	138	1	3	139	0	1
20	196	1	0	96	0	0
21	122	0	0	120	0	1
22	175	0	0	173	0	1
23	56	0	1	58	0	0
24	39	0	1	38	0	0
25	561	1	0	276	0	2
26	116	2	2	111	1	3
27	148	2	1	143	0	0
28	231	1	1	242	0	0
29	89	0	1	88	0	0
30	168	1	1	172	0	0
31	116	0	0	61	0	0
32	1172	1	1	377	0	0
33	706	1	0	325	0	0
34	204	0	1	185	1	2
35	288	1	1	280	0	0
36	254	0	1	272	0	0
37	314	0	1	154	0	0
38	162	0	0	160	0	0
39	442	1	1	112	0	0
40	394	1	1	124	0	0
41	2635	12	15	2634	10	9
42	1456	2	27	2895	5	41
43	101	0	0	51	0	0
44	232	0	0	115	0	0
45	70	0	0	75	0	0
46	25	0	0	24	0	0
47	196	0	0	195	0	0
48	676	0	0	225	0	0

Table S1: Data for the rosiglitazone study. Shown are the study sizes (N), number of myocardial infarctions (MI), and number of cardiovascular deaths (CVD) for the treated and control arms.

2 Computational Details of XRRmeta

Here we detail the three steps involved in implementing XRRmeta: (i) initialization, (ii) iteration, and (iii) correction. Let s denote the step size for the grid along the μ axis and k a positive integer.

Initialization step. Obtain starting points for the iteration step.

Step a. Compute $(\mu_{LB}^{MOM}, \mu_{UB}^{MOM})$, the CI based on the asymptotic χ^2 approximation to $\widetilde{\mu}$.

Step b. Evaluate

$$\tilde{p}(\widetilde{\mu_{LB}^{MOM}}; \mathcal{D}^0) \text{ and } \tilde{p}(\widetilde{\mu_{UB}^{MOM}}; \mathcal{D}^0)$$

where $\tilde{p}(\mu; \mathcal{D}^0) = p\left\{\mu, \nu_{\sup}(\mu); \mathcal{D}^0\right\}$, $\widetilde{\mu_{LB}^{MOM}} = \max(s, \mu_{LB}^{MOM})$, and $\widetilde{\mu_{UB}^{MOM}} = \min(1-s, \mu_{UB}^{MOM})$.

Step c. Take the upper and lower starting values as

$$\widetilde{\mu}_{\inf} = \widetilde{\mu_{LB}^{MOM}} I\left\{\tilde{p}(\widetilde{\mu_{LB}^{MOM}}; \mathcal{D}^0) \geq \alpha\right\} + \widetilde{\mu} I\left\{\tilde{p}(\widetilde{\mu_{LB}^{MOM}}; \mathcal{D}^0) < \alpha\right\}$$

and

$$\widetilde{\mu}_{\sup} = \widetilde{\mu_{UB}^{MOM}} I\left\{\tilde{p}(\widetilde{\mu_{UB}^{MOM}}; \mathcal{D}^0) \geq \alpha\right\} + \widetilde{\mu} I\left\{\tilde{p}(\widetilde{\mu_{UB}^{MOM}}; \mathcal{D}^0) < \alpha\right\}.$$

Iteration step. Iterate along the grid on the μ axis based on the initialization points.

Step a. Find the upper bound by iterating out from $\widetilde{\mu}_{\sup}$ until

$$\mu_{UB}^{\text{it}} = \inf_{\mu}\left[\mu \mid \tilde{p}(\mu; \mathcal{D}^0) \geq \alpha\right].$$

Step b. Find the lower bound by iterating out from $\widetilde{\mu}_{\inf}$ until

$$\mu_{LB}^{\text{it}} = \sup_{\mu}\left[\mu \mid \tilde{p}(\mu; \mathcal{D}^0) \geq \alpha\right].$$

Correction step. Evaluate a (μ, ν) grid beyond the bounds from the iteration step.

Step a. Obtain p values for μ in $(\mu_{UB}^{\text{it}}, \mu_{UB}^{\text{it}} + ks)$ and $(\mu_{LB}^{\text{it}} - ks, \mu_{LB}^{\text{it}})$ and let

$$\mu_{UB} = \max\{\mu_{UB}^{\text{it}} + ks \mid \tilde{p}(\mu_{UB}^{\text{it}} + ks; \mathcal{D}^0) \geq \alpha, k \geq 0\} \text{ and}$$

$$\mu_{LB} = \min\{\mu_{LB}^{\text{it}} - ks \mid \tilde{p}(\mu_{LB}^{\text{it}} - ks; \mathcal{D}^0) \geq \alpha, k \geq 0\}.$$

Step b. Compute $p(\mu; \mathcal{D}^0) = \sup_{\nu} p(\mu, \nu; \mathcal{D}^0)$ for $\mu \in (\mu_{UB}, \mu_{UB} + \delta)$ and $\mu \in (\mu_{LB} - \delta, \mu_{LB})$ for some small $\delta > 0$. For example, for the upper bound, compute p values

along $j = 1, \ldots, J$ equally spaced values of $\mu^j \in (\mu_{UB}, \mu_{UB}+\delta)$ and corresponding $i = 1, \ldots, I_j$ equally spaced values of $\nu^{i_j} \in (0, \nu_{\sup}(\mu))$ to approximate $p(\mu^j; \mathcal{D}^0)$ as

$$\max\{p(\mu^j, \nu^{i_j}; \mathcal{D}^0) \mid i = 1, \ldots, I_j\}.$$

Step c. Compute the upper bound of the interval as

$$\max\{\mu^j \mid p(\mu^j; \mathcal{D}^0) \geq \alpha, j = 1, \ldots, J\}$$

and the lower bound in a similar manner.

3 Additional Simulation Results

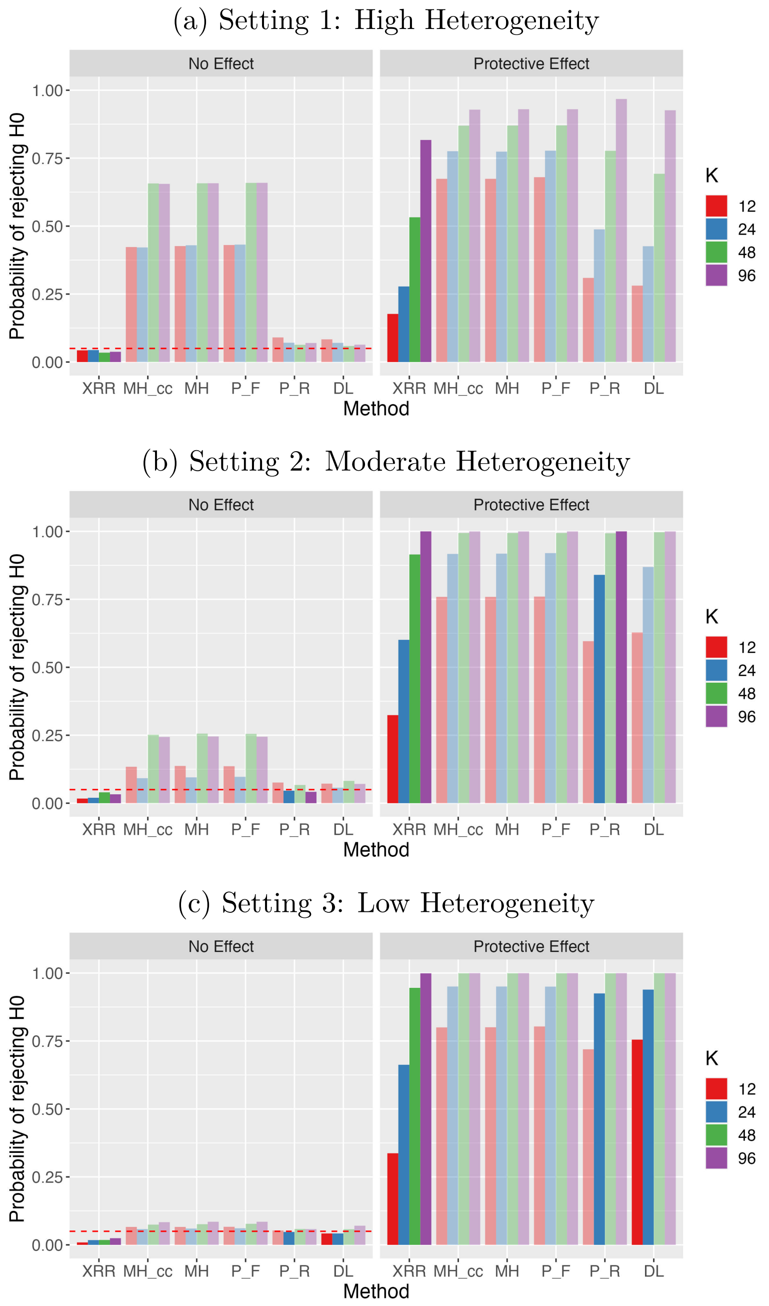

Figure S1: Type I error and power with $r_0 = 0.03$ for XRRmeta (XRR), Mantel-Haenszel with and without a 0.5 continuity correction (MH, MH-CC), the fixed and random effects Peto method (Peto-F, Peto-R), and the DerSimonian-Laird method with a 0.5 continuity correction (DL). Methods that do not control the type I error are shown in a lighter shade.

4 Face Mask Data

	Face Mask		No Face Mask	
Study ID	N	Transmission Events	N	Transmission Events
1	16	3	15	4
2	123	8	354	43
3	98	11	115	61
4	202	46	55	31
5	24	3	4	2
6	7	0	2	1
7	31	0	6	3
8	43	8	72	17
9	61	17	18	14
10	42	8	25	14
11	23	3	9	5
12	278	0	215	10
13	51	0	203	13
14	1286	1	4036	119
15	116	6	101	12
16	62	2	10	2
17	26	3	60	33
18	27	6	71	39
19	218	0	230	6
20	444	1	308	16
21	42	0	6	0
22	24	0	10	0
23	60	0	45	0
24	13	0	19	0
25	64	0	13	0
26	61	0	1	0
27	89	12	98	25
28	146	25	229	69
29	9	0	154	7

Table S2: Data for the face mask study. Shown are the study sizes (N) and the number of transmission events for the face mask and no face mask arms.

5 Example analysis with XRRmeta

Below we provide an example analysis of the Rosiglitazone data to illustrate the implementation of XRRmeta in practice.

```
# Read in and format the data.
my_data <- read.csv("rosiglitazone.csv")

n1 <-  my_data[, "n.t"]
e1_mi <- my_data[, "e.mi.t"]
e1_cvd <- my_data[, "e.death.t"]

n2 <-  my_data[, "n.c"]
e2_mi <- my_data[, "e.mi.c"]
e2_cvd <- my_data[, "e.death.c"]

mi_data <- cbind(n1, e1_mi, n2, e2_mi)
cvd_data <- cbind(n1, e1_cvd, n2, e2_cvd)
colnames(mi_data) <- colnames(cvd_data) <- c("size_1", "events_1",
                                             "size_2", "events_2")
# Load the XRRmeta package.
library(RareEventsMeta)

# Run analysis with XRRmeta.
mi_xrrmeta <- ExactConfInt(
  events_1 = mi_data_dzr[, "events_1"],
  size_1 = mi_data_dzr[, "size_1"],
  events_2 = mi_data_dzr[, "events_2"],
  size_2 = mi_data_dzr[, "size_2"],
  reps = 2000,
  step_size = 0.001,
  maxit = 500,
  mu_extra_steps = 10,
  nu_extra_steps = 10
)
```
